# Evaluation of the Regulatory Review Process of the Zambia Medicines Regulatory Authority: Challenges and Opportunities

**DOI:** 10.1007/s43441-024-00730-6

**Published:** 2024-12-27

**Authors:** Constance Sakala Chisha, Makomani Siyanga, Stephanie Leigh, Adem Kermad, Stuart Walker

**Affiliations:** 1 University of Witwatersrand, 7 York road, Parktown, Johannesburg, 2017 South Africa; 2 Zambia Medicines Regulatory Authority, P. O Box 31890, Lusaka, Zambia; 3 Centre for Innovation in Regulatory Science, 70 St Mary Axe, London, EC3A 8BE UK

**Keywords:** Zambia Medicines Regulatory Authority (ZAMRA), Regulatory review models, Key milestones, Good review practices, Quality decision-making process

## Abstract

**Purpose:**

This study aimed to assess the current regulatory review process of the Zambia Medicines Regulatory Authority (ZAMRA) by identifying the key milestones and target timelines achieved for products approved from 2020 to 2023, as well as good review and quality decision-making practices implemented in the review process.

**Methods:**

A standardised, validated questionnaire; Optimising Efficiencies in Regulatory Agencies (OpERA) and the OpERA Data Collection Template were completed by the author.

**Results:**

Three review models are used by ZAMRA to review new active substances (NASs) and generic products: verification, for products prequalified by the World Health Organization or approved by a stringent regulatory authority (SRA); abridged, for well-established molecules or SRA-approved products; or full, for products not otherwise prequalified. Good review practices and quality decision-making processes were followed but could be improved.

**Conclusion:**

This study assessed the overall ZAMRA operation and identified the key milestones in the review process for products approved from 2020 to 2023, target timelines achieved and the compliance to standard good review and quality decision-making practices.

## Introduction

### Zambia Medicines Regulatory Authority

Zambia is a large, landlocked country located in the Southern part of Africa, covering a total area of 752,612 square kilometres [1]. It shares its borders with eight countries, namely Angola, Botswana, Democratic Republic of Congo, Malawi, Mozambique, Namibia, Tanzania and Zimbabwe [2]. As of September 2022, Zambia was considered to have a large population, comprising 19,610,769 citizens [3] with a gross domestic product (GDP) of 29.16 billion US dollars in 2022, which is projected to grow by 4.2% by the end of 2024 [4].

The Zambia Medicines Regulatory Authority (ZAMRA) is the statutory body that is mandated by law to regulate and control the manufacture, importation, exportation, possession, storage, distribution, supply, promotion, advertising, sale and use of medicines and allied substances, and the control of clinical trials and conduct of quality testing in Zambia [5–7]. Further, it provides for the control and restrictions relating to medicines and other substances including disinfectants, food supplements and feed additives [5]. Medicine regulation in Zambia dates back to 1940 when the Pharmacy and Poisons Act, Chapter 299 of the Laws of Zambia was first enacted [8]. Since its inception, the Act has undergone various repeals, with the current version being the Medicines and Allied Substances Act of 2013 which provides for the formation of ZAMRA [9]. The Authority has improved its regulatory systems in as far as registration of medicines is concerned [10]. To ensure harmonisation of registration standards with the global standards, ZAMRA adopted the International Council for Harmonisation of Technical Requirements for Pharmaceuticals for Human Medicines (ICH) guideline M4 on Common Technical Document (CTD) format [10]. This has made it easier for applicants to prepare the dossiers, as the same format is submitted to different countries without reformatting the technical information [11]. In its quest to improve the quality management system ZAMRA has introduced the Integrated Regulatory Information Management System (IRIMS) which allows applicants to submit and track applications electronically [12]. Zambia is a founding member of the ZAZIBONA initiative that comprises the countries in the Southern African Development Community (SADC), that is, Zambia, Zimbabwe, Botswana, Namibia, South Africa, Tanzania, Mozambique, Malawi and the Democratic Republic of Congo [13]. This collaboration aims to shorten assessment time and aids in building capacity amongst assessors and inspectors, thus improving access to medicines [14].

Strengthening regulatory systems for medicines is critical for a well-functioning health system to achieve universal health coverage [15]. Strengthened regulatory systems can contribute to the social and economic development of the country by supporting the manufacturing of medicines; however, many low- and middle-income countries (LMICs) lack the capacity to effectively and efficiently regulate medicines [16]. This is because the establishment and maintenance of mature regulatory systems is a highly resourced venture [17]. To help strengthen regulatory systems globally, the World Health Organization (WHO) developed the Global Benchmarking Tool (GBT) in 2014 as part of its capacity-building programme [18, 19]. This tool was developed to allow the WHO and national medicines regulatory authorities (NMRAs) to evaluate and analyse evidence on performance, and facilitate the formulation and implementation of institutional development plans (IDPs) for a regulatory function [20]. Most NMRAs, especially in LMICS are operating with some level of regulation, but not always up to the required standard [21].

Assessing the current performance of ZAMRA will help in identifying its strengths and weaknesses as far as the review process is concerned. This will in turn help improve its regulatory systems as it aims to attain WHO GBT maturity level 3 (indicating an established, quality-assured regulatory system). The aim of this study was therefore to assess the current regulatory performance of ZAMRA with the aim of identifying its strengths and weaknesses.

### Objectives

The objectives of this study were to assess the current ZAMRA regulatory review process by

1. Identifying the key milestones and target timelines achieved for products approved from 2020 to 2023;
2. Evaluating the overall performance of the review models and different types of products approved during the period 2020 to 2023;
3. Assessing the authority’s compliance with good review practices and quality decision-making practices employed in the review process; and
4. Identifying challenges and opportunities for an enhanced regulatory review process in Zambia.

## Materials and Methods

### Ethical Approval

The study was granted ethical approval by the Human Research Ethics Committee (Medical) of the University of Witwatersrand, Johannesburg, South Africa (Waiver number: H24/01/05).

Permission was granted by the Zambia Medicines Regulatory Authority for the collection of data and for its subsequent publication.

### Study Rationale

The performance of the regulatory review process of ZAMRA to date has not yet been assessed; therefore, this study will serve as the benchmark for future evaluations.

### Data Collection Process

The regulatory review process of ZAMRA was assessed using the validated Optimising Efficiencies in Regulatory Agencies (OpERA) questionnaire, developed by the Centre for Innovation in Regulatory Science (CIRS). The OpERA project was initiated in 2013 based on requests from regulatory agencies [22]. This questionnaire is a unique regulatory-strengthening tool that enables all critical information necessary to assess a regulatory authority’s performance to be documented systematically [23].

The questionnaire was used to map the key milestones and activities associated with the review process and practices within ZAMRA. The questions were completed by the principal investigator (a senior assessor at ZAMRA) and were then verified by the Director General of the Zambian agency.

The questionnaire consists of six main parts [23, 24]:

- **Part 1: *Organization of the Authority*** documented the information on the current structure and size of the Authority, and resources of the Authority.
- **Part 2: *Types of review models*** identified the different types of review model(s) used for the scientific assessment of medicines in terms of the data assessed and level of detail by the Authority, as well as how the Authority might implement a reliance strategy based on the results of assessments and reviews carried out by a reference agency.
- **Part 3: *Key milestones in the review process*** captured the main steps in the review and approval process and identified key milestone dates using the OpERA Data Collection Template, which mapped the process of assessment starting from receipt of the dossier, through validation/screening, the number of cycles of scientific assessments including the questions to the sponsor/applicant and expert registration committee meetings, to the final decision on approval or refusal of a product for registration. A standardised process map embedded in the questionnaire was based on the experience of studying established and emerging regulatory authorities. Data for NASs, biologicals, vaccines and biosimilars as well as generics for human use registered by the ZAMRA during the period 2020–2023 were collected. These data were sourced directly from the Unit within the Authority responsible for the regulatory review process.
- **Part 4: *Good review practices (GRevP)*** evaluated how quality was built into the regulatory process by examining activities that had been adopted to improve consistency, transparency, timeliness and competency.
- **Part 5: *Quality decision-making processes*** explored the quality of the Authority decision-making practices and measures in place to ensure that quality decisions were made around the data during the registration process.
- **Part 6: *Concluding Observations*** provides the perception of ZAMRA regarding its unique positive qualities and the major challenges it faces in carrying out the review of new marketing authorisations and making them available to meet patients’ needs.

## Results

The results are presented in six parts: Part 1-Organisation of the authority; Part 2-Types of review models; Part 3-Key milestones in the review process; Part 4-Good Review Practices building quality into the regulatory process; Part 5-Quality decision-making practices and Part 6-Concluding observations, with a summary of the challenges and opportunities in the Zambian regulatory review.

### Part 1—Organisation of the Zambia Medicines Regulatory Authority (ZAMRA)

The ZAMRA is a statutory body under the Ministry of Health with a total staff of 151. It has its presence in six areas including Lusaka, Copper belt, Eastern, North-Western, Northern, Muchinga and Southern provinces. At the time of the study, the Authority had a total of 14 reviewers, 9 of whom are pharmacists and 5 who are scientists. An additional 10 reviewers (7 pharmacists and 3 scientists with medical background) were employed by the Authority through a cooperating partner to assist with the backlog of applications.

**Figure 1.**
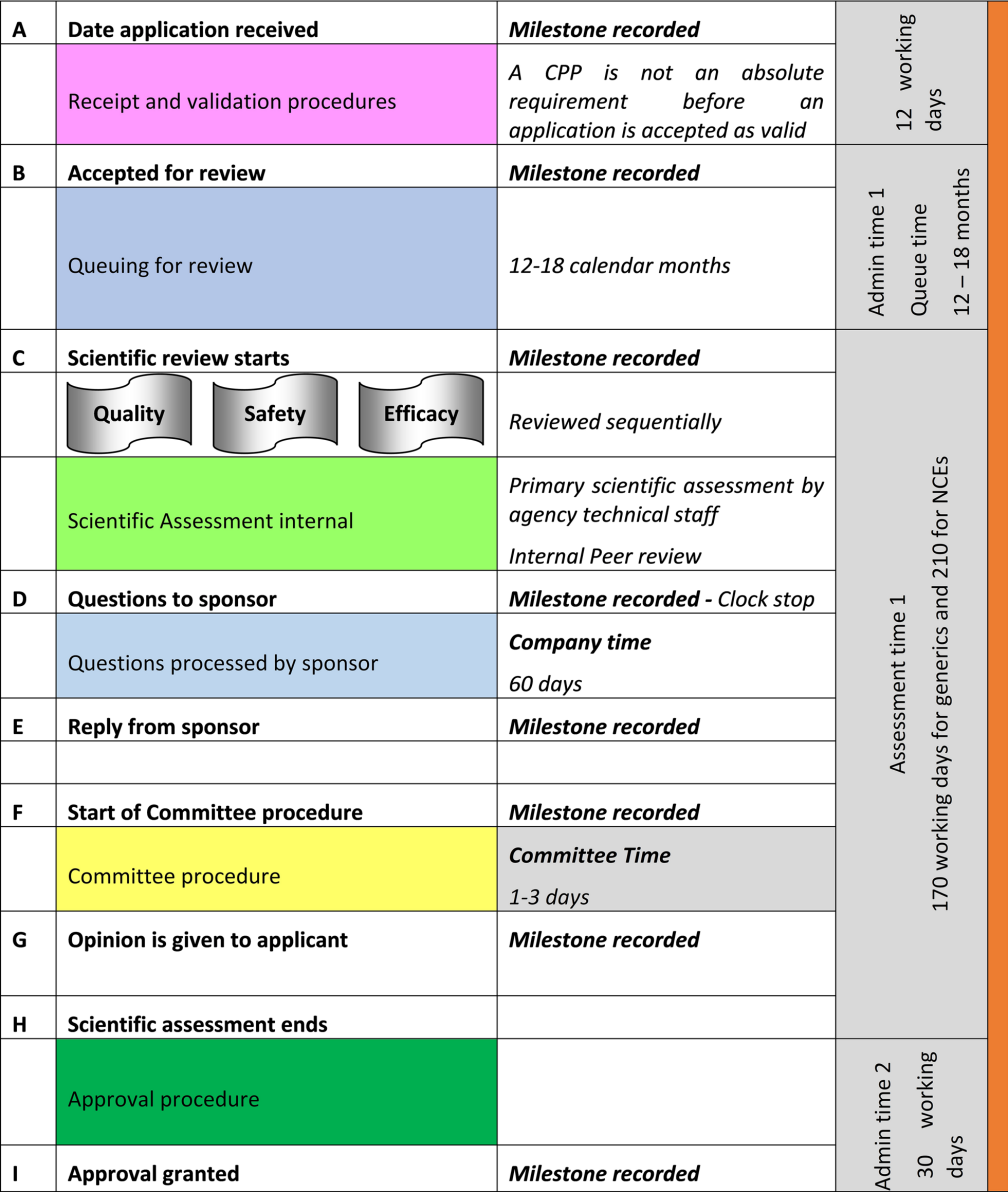
Regulatory Review Process Map for Zambia Showing Target Times In Calendar Days; Representing the Review and Authorisation of a Product that Goes to Approval After One Review Cycle.

The certificate of pharmaceutical product (CPP) is a requirement in the submission for the grant of marketing authorisation. The Authority has set target timelines for NAS applications including the overall time for the review and the approval of an application. Once the assessment is carried out, questions to sponsors are submitted in batches at fixed points in the review procedure and applicants are given a specified timeframe within which to respond to the questions posed. The Authority recognises medical urgency as a criterion for accelerating the review and approval process for qualifying products. Because inadequate staffing levels preclude parallel reviews, the review of the different sections of the technical data (product information, quality, safety and efficacy) are carried out sequentially by the same assessors (Fig. 1).

Zambia does not regulate the pricing of commodities, including medicines; therefore, the Authority does not hold price negotiations during the review process. Sample analyses do not impede marketing authorisation as the Authority relies of the certificate of analysis generated from the finished product manufacturer.

The Authority is endeavouring to build quality into the review process and has put measures in place to monitor and improve quality and ensure consistency and transparency. ZAMRA is ISO 9001_2015 certified therefore it meets the requirements of the standard. The basis for implementing a QMS is using continuous improvement. A systematic auditing provides the identification for opportunities of improvement and risk management as the QMS is regarded as organic since it undergoes changes based on the identified needs. Standard operating procedures are employed and assessment reports are used to standardise the content and format of the evaluation reports.

In 2021, the Authority launched IRIMS, through which applications are submitted and processed electronically. Since its inception, both the Authority and the applicant have been able to monitor the progress of the application from submission to granting of marketing authorisation.

### Part 2—Types of Review Models

ZAMRA conducts the three types of established review models including verification, abridged and full review. An additional fast track route is used for priority medicines to ensure the rapid accessibility of medicines to patients within the shortest possible timeframe.

#### Verification Model

This type of review model is used by ZAMRA for products that have been prequalified by the WHO or approved by stringent regulatory authorities (SRAs). The applicant must demonstrate sameness of the dossier submitted with that reviewed and prequalified by WHO. The Authority does not review the submission but rather verifying sameness of the dossier with that submitted and prequalified by WHO.

#### Abridged Model

This model is used for products that were submitted before 2015. The abridged template was used in the review process. In 2015, the Authority adopted the Quality Overall Summary as the evaluation template following the adoption of the Common Technical Document (CTD) format. This method of review is mainly used for generic products.

#### Full Review Model

The Authority conducts a full review of applications including NASs and generics that includes assessment of the product information (summary of product characteristics, patient information leaflet and labels) quality, safety and efficacy data of the dossier, which is submitted in the CTD format. The Quality Overall summary is used as the assessment template.

#### Priority/Fast Track Model

Fast track review model is used for products that are of high public health concern. This is an expedited model in which applications are reviewed within 90 working days. The dossier is submitted in the CTD format.

### Part 3-Key Milestones in the Review Process

The process map of the review process by ZAMRA is given in Fig. 1. This map provides a simplified representation of the main steps taken in the review of an NAS and shows the review and authorisation of a product that is approved on the first cycle. It excludes those that are approved after a second cycle of questions or queries. Rejection and appeals are not included in the process map and the target timelines are given in calendar days (Fig. 1).

#### Validation Phase

The application is screened and validated within 12 working days. The following parameters are reviewed and verified during screening: the legal status of the applicant and the local responsible person, good manufacturing process (GMP) status of the manufacturer, the application fees paid, and the application format (CTD). Since dossiers are submitted online, samples are submitted separately. Applications are screened to verify completeness of the submission. The applicant is given a period of 60 days to respond to questions raised at screening. Once an application is validated, it enters the queue for review and applications are reviewed on a first-come-first-served basis.

#### Evaluation Phase

Once applications are screened and validated, they are held in a queue for a period of approximately 12 to 18 months although priority products are always taken out of the queuing system and assessed immediately. The Authority has qualified technical experts who conduct sequential scientific assessments of the product information, quality, safety and efficacy of applications. The Authority does not conduct meetings with applicants to discuss the questions and queries during the assessment process. The questions raised are sent to the applicants after the initial assessment and they are given 60 days within which to respond to the questions, which time a “clock stop” is applied. This means that the time it takes for the applicant to respond to the queries is not counted as the regulatory time. After two rounds of queries, the application is considered by the Expert Committee known as the Technical Committee on Human Medicines, which meets bimonthly and will recommend either rejection or approval of the application.

#### Decision Phase

Marketing authorisation is dependent on sample analysis and GMP inspection is not carried out in parallel with the assessment. The Authority relies on manufacturer’s analysis and GMP inspections conducted by recognised regulatory Authorities and WHO. The Authority grants the marketing authorisation (MA) certificate upon completion of the review process. If the applicant fails to address all the queries raised after being given two changes to respond their application is rejected.

#### Metrics of Applications Registered 2020 to 2023

The Authority approved a total of 424 products from 2020 to 2023, 118 of these products were approved in 2020, while 142, 87 and 77 products were approved in 2021, 2022 and 2023, respectively (Table 1). There was a reduction in the number of applications approved in the years 2022 and 2023. As seen in Table 1, most of the products registered in Zambia were generics, as they are less expensive as compared with branded products, a trend similar across Africa as seen in the Africa Generic Pharmaceutical Market Size, Share & Trends Analysis Report of 2023 to 2030 [25].

**Table 1:** Characteristics of Products Approved by ZAMRA (2020–2023).

Characteristic		Year of Approval				
		2020	2021	2022	2023	2020–2023
Overall		118	142	87	77	424
Compound type*	New active substance	3	6	5	8	22
	Generic	109	136	82	64	391
	Biosimilar	0	0	0	5	5
	Vaccine	5	0	0	0	5
Review type	Verification	3	4	7	12	26
	Abridged	62	87	40	41	230
	Full	53	51	40	24	168

*For one approval, compound type was unknown.

#### Characteristics of NASs Approved 2020–2023

During the period 2020 to 2023, the Authority approved a total of 22 NASs, 5 of which were biologicals and 17 pharmaceuticals. In 2020, 3 NASs were approved, 6 in 2021, 5 in 2022 and 8 in 2023. All of them were approved by the Stringent Regulatory Authority (SRA) approved. Of the NASs approved in 2023, 6 (75%) were biologicals while 2 (25%) were pharmaceuticals. There was an increase in the number of submissions and approvals of NASs in 2023 as compared with previous years. Further, the Authority has specialised officers reviewing these products, resulting in an increase in the number of approved products from 2020 to 2023. These are officers who are primarily qualified in Biotechnology and pharmacology, which equip them to review novel applications. A full review was used in the assessment of all the NASs approved (Table 1).

#### Characteristics of Generic Products Approved Between 2020 and 2023

During the period 2020 to 2023, a total of 391 generic products were approved by the Authority (Table 1). Twenty five (25) of these were WHO prequalified. In 2020, 109 products were approved, 136 in 2021, 82 in 2022 and 64 in 2023. There was a reduction in the number of products approved in 2021 and 2022 due to the COVID-19 pandemic and the introduction of the IRIMS, which slowed the review process during this period.

#### Overall Decision Timelines for Approved NASs

**Figure 2.**
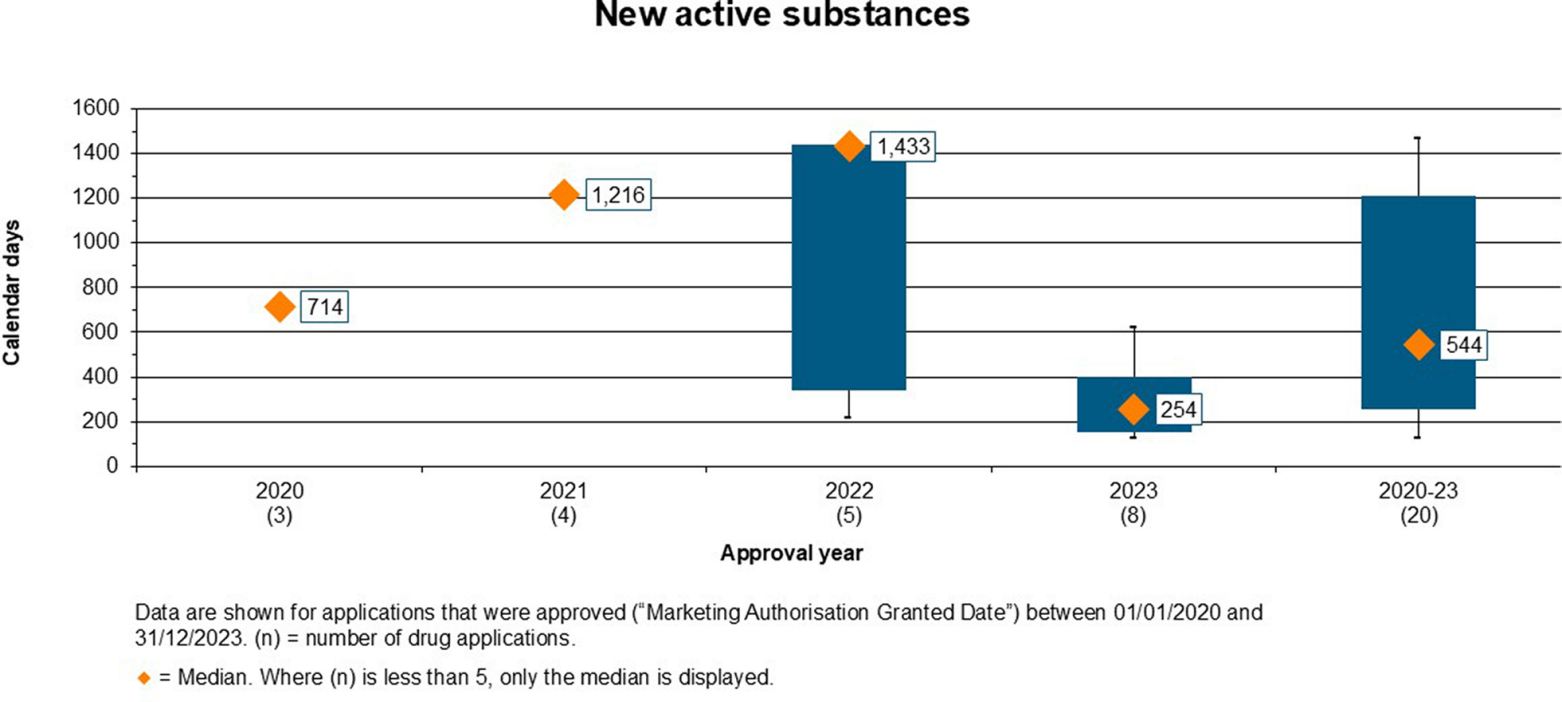
Overall Approval Time of the New Active Substances Approved by ZAMRA (2020–2023).

**Figure 3.**
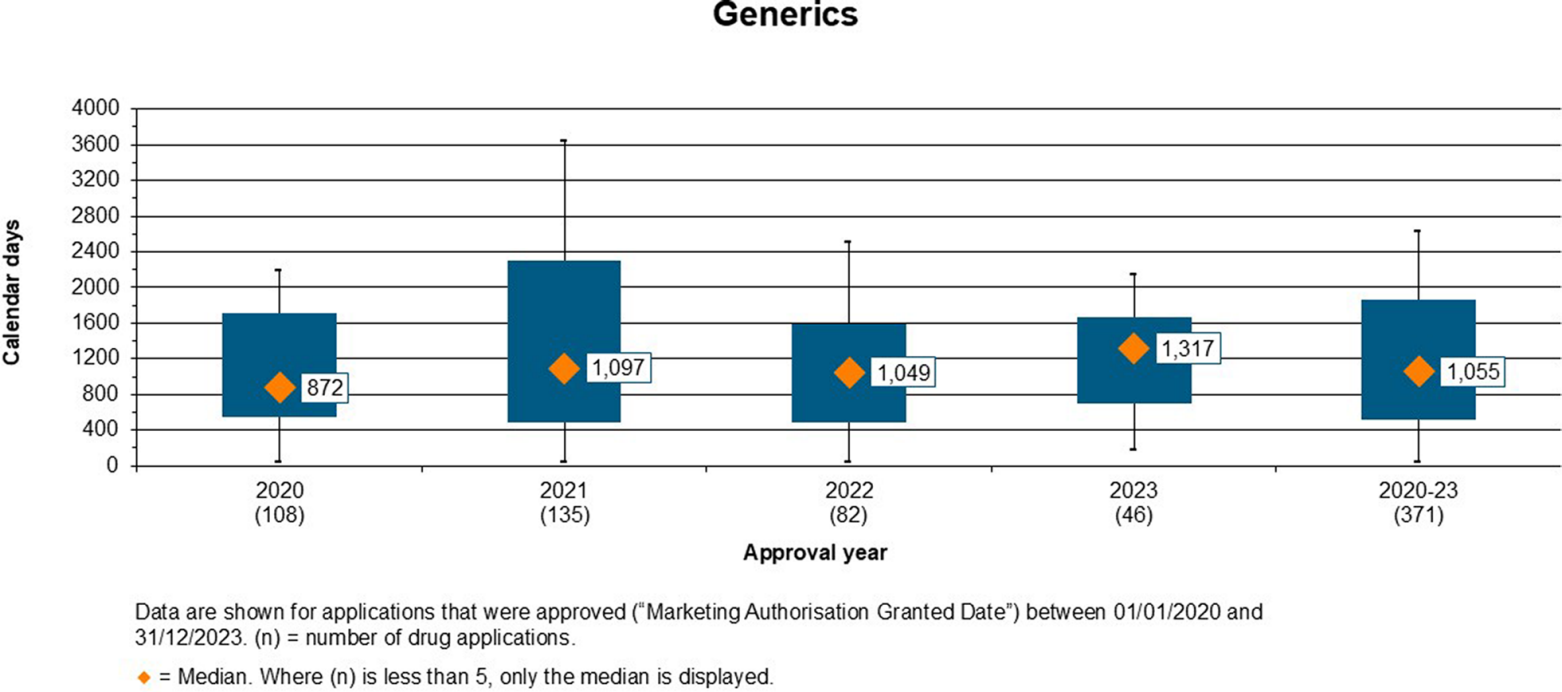
Overall Approval Time of Generics Approved by ZAMRA (2020–2023).

The overall timelines for approved products from 2020 to 2023 is given in Fig. 2 (NASs) and Fig. 3 (Generics). The diamond represents the median value, the box represents the range between the 25th and the 75th percentile, while the whiskers represent the outliers, which are the 5th and 95th percentiles. However, the boxes and the whiskers are only shown where n > = 5, and then where this condition is not met the median only is shown. The median approval time for NASs approved between 2020 and 2023 was 544 calendar days, as shown in Fig. 2. The median approval time was 714 calendar days in 2020, 1,216 days in 2021, 1,433 calendar days in 2022 and 254 calendar days in 2023. It was observed that the approval times were longer in 2021 and 2022, as compared to the years 2020 and 2023, due to the COVID-19 pandemic, which slowed the review process and delayed submission of responses by applicants. In the year 2023, the median approval time for NAS improved significantly due to the easing of COVID-19 pandemic pressures.

#### Overall Approval Time for Generics

The overall approval time for generics from 2020 to 2023 was 1,055 calendar days (Fig. 3). The approval time was 872 days in 2020, 1,097 days in 2021, 1,049 days in 2022 and 1,317 days in 2023. The approval times are extended as they included the time allocated to the applicant for review of comments made by evaluators/assessors. They were cumulative meaning they included the regulator and applicant time. Applicants were found to take longer than the assigned time of 120 days to respond to comments and questions.

#### Overall Approval Time as Per Review Model

**Figure 4.**
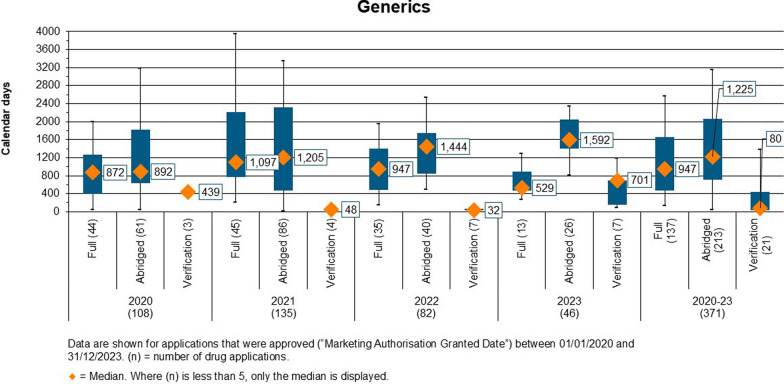
Overall Approval Time of Generic Products by Review Model and Approval Year.

Full review and verification models were used in the assessment of NAS and generic products. Overall, the median approval time for NAS was 592 days using full review and 372 days using the verification model. Figure 4 provides the approval times for each type of review model for generics.

**Figure 5.**
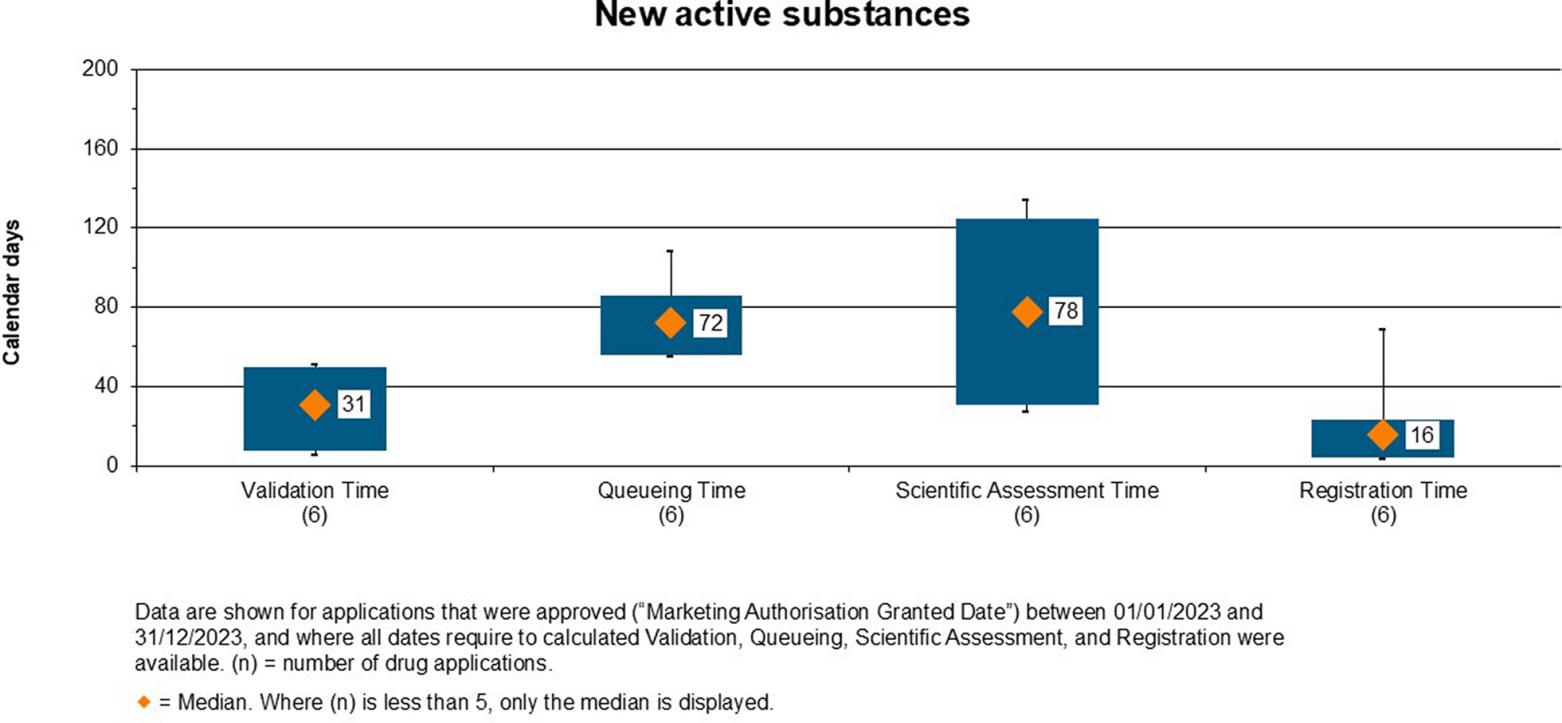
Validation, Queueing, Scientific Session and Registration of New Active Substances Approved in 2023.

The ZAMRA has set target timelines for the validation, queuing and client review times, as per the type of product under review. The study further investigated the timelines for approved NAS in the year 2023. As shown in Fig. 5, it was observed that for NASs approved in 2023 the median validation time was 31 calendar days, the median queueing time was 72 days, the median scientific assessment time was 78 days and the median time for approval by the committee (‘registration time’) was 16 days. The timelines were shorter in 2023 as compared to the overall median approval times from 2020 to 2023.

**Figure 6.**
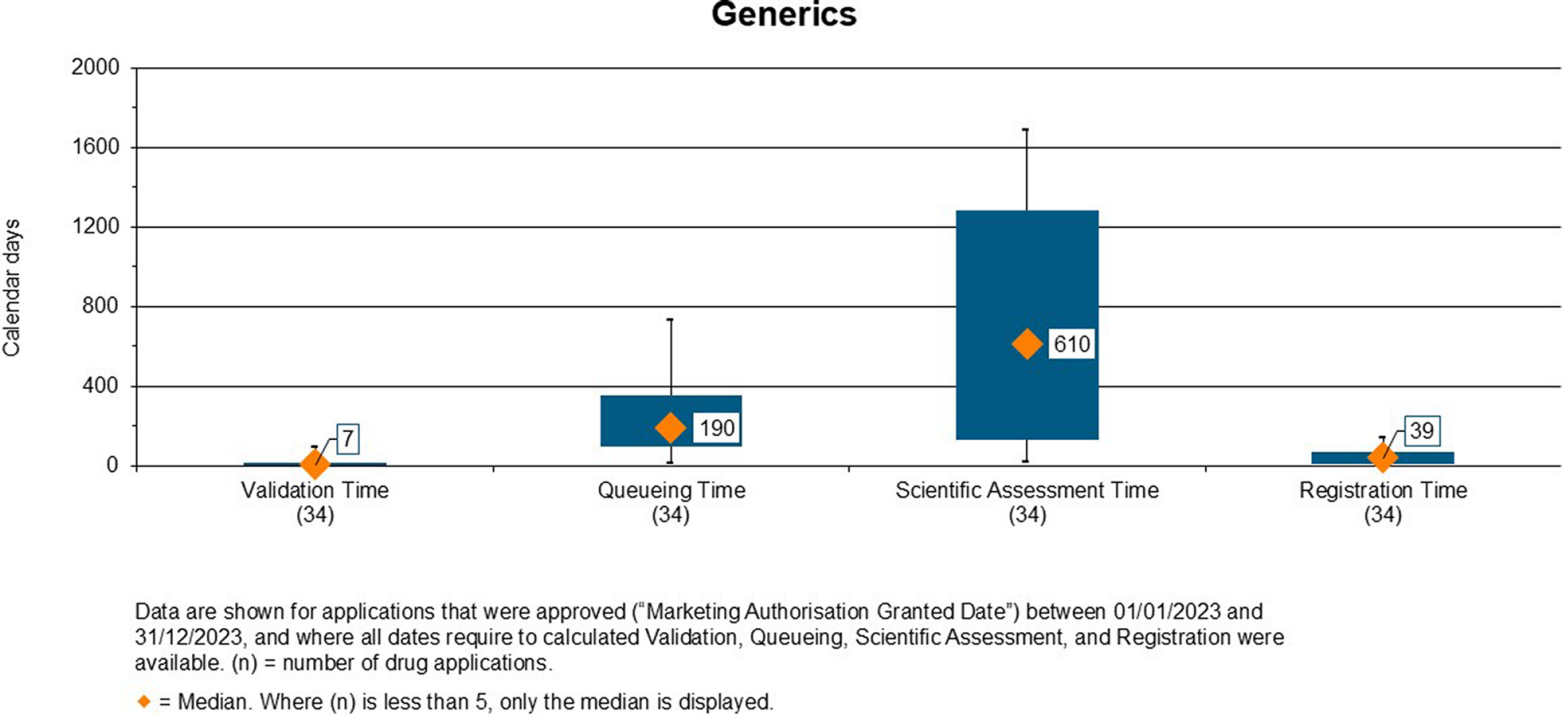
Validation, Queueing, Scientific Assessment and Registration of Generics Approved in 2023

**Figure 7.**
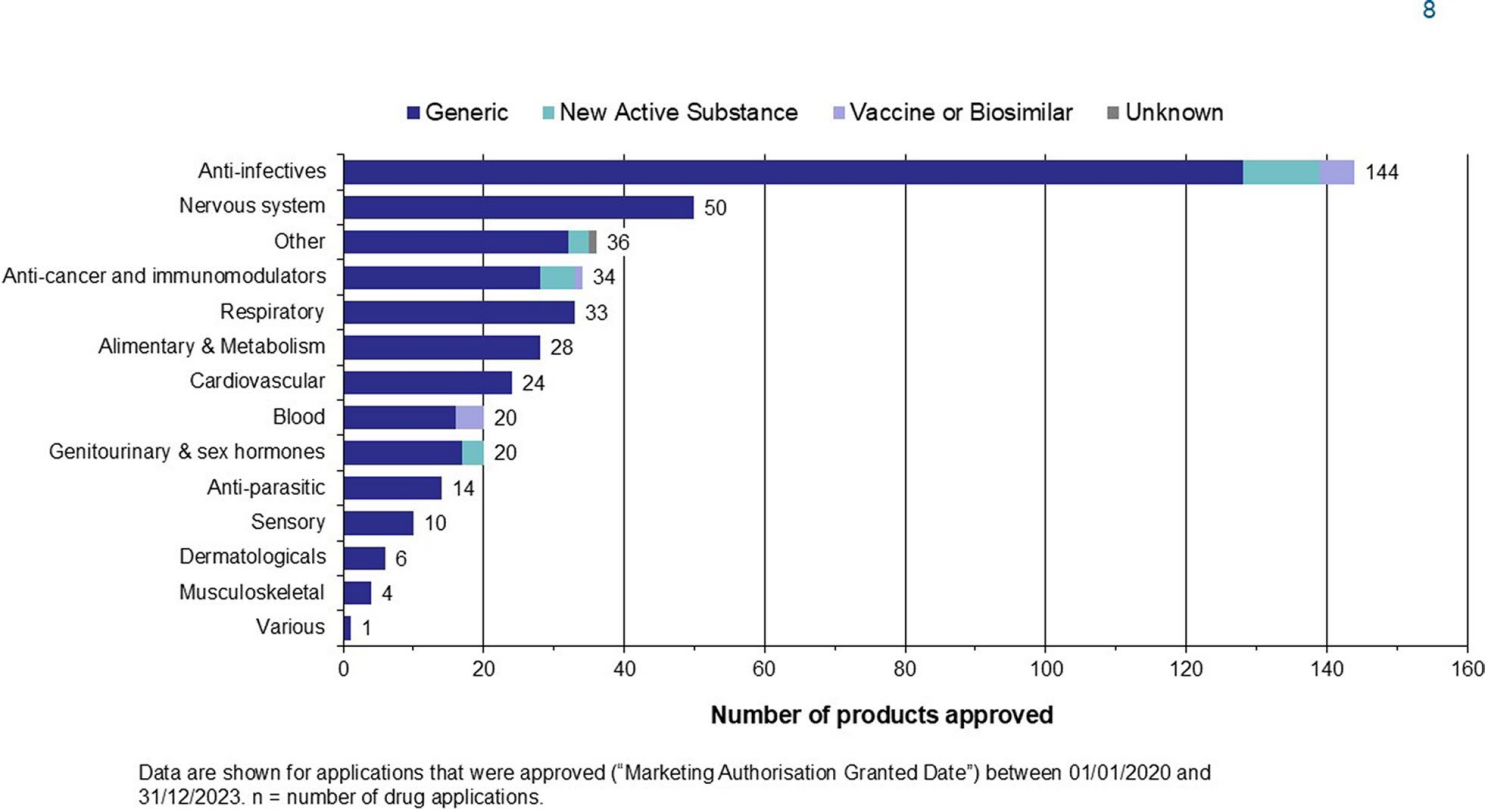
Therapeutic Area of Products Approved by Compound Type (2020–2023).

Figure 6 shows the timelines for generic products approved in 2023. The median validation time was 7 calendar days, the median queuing time was 190 days, scientific assessment time was 610 days and median registration time was 39 days. As shown in Fig. 7, the Authority receives more generics as compared with NASs, as Zambia is a low-middle income country whose source of medicines is mostly India. Anti-infectives, analgesics and anti-cancer drugs were amongst the top three therapeutic areas for approved products in 2023.

#### Overall Approval Time by Therapeutic Area for NASs

During the period under review, the Authority approved products in several therapeutic areas, with the main ones being analgesics, anticancer, immunomodulators and anti-infectives. (Fig. 7). Zambia has seen an increase in the number of various cancers being diagnosed [26], creating a high demand for anti-cancer drugs for use amongst the Zambian population and an increase in the number of new anti-cancer drugs being approved by ZAMRA.

The median approval time for these products were 727 days and 619 days in 2020 and 2023, respectively.

As shown in Fig. 7, during the period 2020 to 2023, 34% (144) of approved products were anti-infectives, 9% (20) were blood and blood forming organs, 8% (34) were anti-cancer and immunomodulators, 8% (33) were respiratory, while 12% (50) were nervous system agents. Zambia, like any other African country, has a high burden of communicable diseases such as tuberculosis, HIV and malaria [27]. This is reflected in the high number of anti-infectives being approved as compared to other therapeutic areas.

### Part 4—Good Review Practices (GrevP)—Building Quality into the Regulatory process

ZAMRA has implemented some quality measures in the review and authorisation of medicinal products, as summarised in Table 2. These documents are available to the public by means of the ZAMRA website.

**Table 2:**
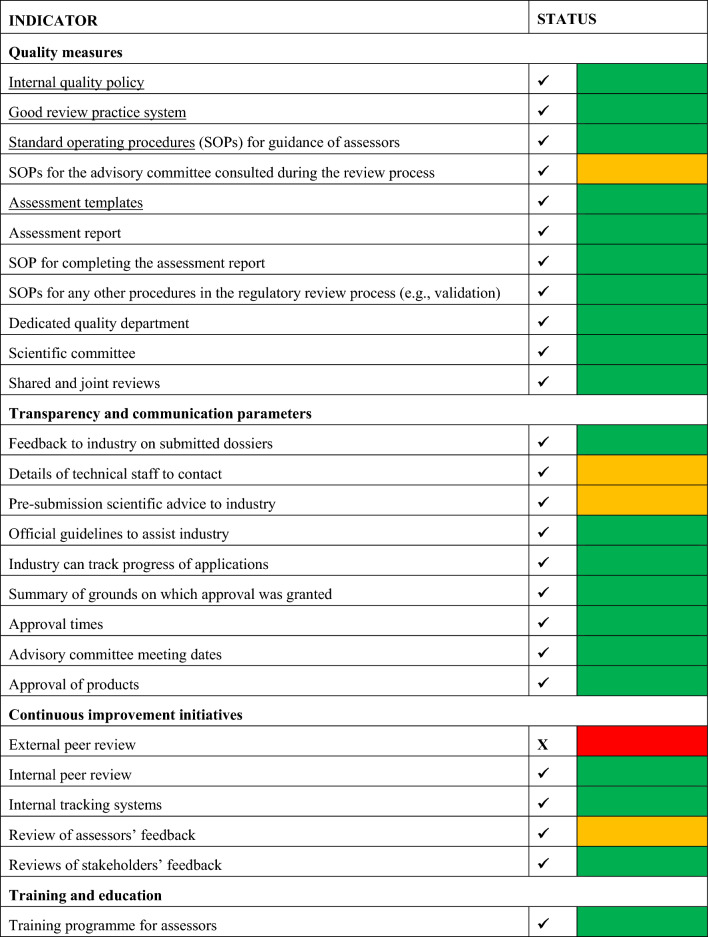

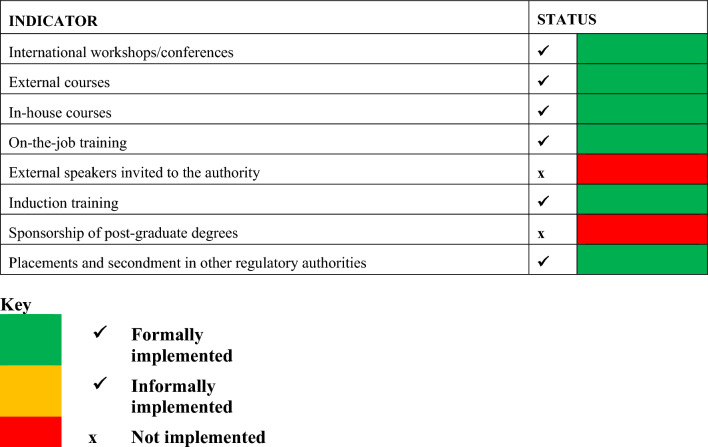
Good Review Practices Implemented by ZAMRA.

#### Quality and Transparency

ZAMRA has identified efficiency, consistency, and improved communication as the three most important reasons for the introduction of quality measurements. Currently, the Authority has a dedicated Unit in place that ensures that quality in the assessment process is maintained. The Authority is committed to continuously improve services to applicants through the establishment of the Quality Management System (QMS) based on the ISO 9001: 2015. ZAMRA is ISO 9001:2015 accredited by the Zambia Bureau of Standards (ZABS). This has helped to strengthen the regulatory system through improvements in the people processes and the services of the Authority [28].

#### Continuous Improvement Measures

The Authority recognises the importance of continuous staff improvement through training to ensure harmonised and high-quality standards for assessments. The Authority conducts induction training and in-house courses, which coupled with international training cover all key areas of health product regulations and are beneficial to the Authority as they increase productivity, competitiveness, sustainability and improved services to the applicants [16]. The Authority participates in the ZAZIBONA SADC harmonised registration initiative. ZAMRA conducts joint assessment with other regulatory Authorities within the SADC region. This has a positive impact on the quality of the assessments by the assessors as it has served as a platform for various training in the assessment of quality and efficacy parts of the dossier. ZAMRA is part of the WHO Collaborative Review Process (CRP) which has assisted in reducing the workload on the Authority and to shorten the review process. The Authority does not sponsor post-graduate degree studies for its staff and currently does not conduct external peer reviews when an NAS application is assessed, and there are no plans to introduce these within the next two years.

### Part 5—Quality Decision-Making Practices

The decision to approve or reject an application for the grant of marketing authorisation is based on the framework that is well defined. The Authority is implementing the quality decision–making practices as shown in Table 3; however, currently it does not conduct any training in decision- making processes.

**Table 3:** ZAMRA Quality Decision-Making Practices.

Practice	Implemented into Framework			Adhered to in Practice		
	Fully	In Progress	Not Implemented	Fully	In Progress	Not Implemented
Have a systematic, structured approach	✔			✔		
Assign clear roles and responsibilities	✔			✔		
Assign values and relative importance to decision criteria	✔			✔		
Evaluate both internal and external influences/biases		✔			✔	
Examine alternative solutions		✔			✔	
Consider uncertainty		✔			✔	
Re-evaluate as new information becomes available	✔			✔		
Perform impact analysis of the decision			✔			✔
Ensure transparency and provide a record trail	✔			✔		
Effectively communicate the basis of the decision	✔			✔		

### Part 6—Concluding Observations

The electronic management system has enhanced the tracking of applications by the Authority as well as the applicants; however, the Authority needs to set target timelines for each milestone in the review process based on the type of application. With continuous improvement and monitoring review timelines can be reduced, but in order for the quality of the submission to be improved, there is a need to conduct training in dossier compilation. The Authority has engaged external assessors to assist in the assessment and also reduction of the extended timelines.

## Discussion

The assessment of the performance of a NMRA is a key process in benchmarking as it aids in identifying Authority gaps and weaknesses [18]. ZAMRA, like any other NMRA in a LMIC, is facing similar challenges of inadequate staffing and increased volumes of applications for marketing authorisation. In addition, the increasing complexity of submissions make it difficult to meet the expected assessment timelines, affecting patients’ timely accessibility [29, 30]. With less than 20 assessors appointed to the ZAMRA, it is difficult to evaluate products and grant marketing authorisation within set timelines.

From this study it was observed that ZAMRA approved more generics than NASs, with more than 90% of the products approved in the period under review being generics. The use of generic products has been increasing globally as a result of economic pressures and the expiry of patents on the widely used medicines [31]. Generic medicines are less expensive than the branded medicines making them affordable for many patients in LMICs who in most cases do not have access to health insurance [32]. A study conducted by the Competition and Consumer Protection Commission (CCPC) in 2022 showed that about 83% of the medicines on the Zambian market were generics with 17% being originator medicines, consistent with the results of this study.

Regardless of the review model, regulatory timelines are longer for generics than for NASs. The results of this study pointed to the need for different timelines for NASs and generics based on differences in reviewed information and the Authority has now set specific timelines based on the type of product reviewed. In this study, the median approval time for generics was 1,055 calendar days and 544 calendar days for NASs. These were half the timelines for the former Medicines Control Council (MCC) of South Africa from 2011 to 2017 (2092 calendar days) but with continuous monitoring and the introduction of the risk-based assessment the review timelines in South Africa were reduced to 511 calendar days [33]. It is clear from the data obtained that the timelines are extended and therefore should be reduced to acceptable and competitive timelines. Continuous efforts and strategic initiatives are being implemented to significantly reduce these review timelines by introduction of other regulatory modalities such as use of reliance and recognition to avoid duplication of work carried out by the recognised NMRAs. Though the Authority is implementing a risk based approach in the review process as has been demonstrated through the use of the WHO collaborative procedure and work sharing through Zazibona joint assessments there is need to have well defined registration pathways using the electronic management system. This will help improve efficiency and effective of the review process thereby significantly reducing the extended timelines. With limited resources and capabilities, it is important for the Authority to implement a well defined risk based approach in the review of medicines for marketing authorization. Having clear registration pathways for SRA approved, WHO prequalified products, those reviewed through joint assessments or work sharing, normal reviews and fast track will enable applicants selected the appropriate pathway. With each pathway having its set timelines will shorten the timelines as each pathway will be monitored separately.

A study conducted by Sithole (2021) assessed the approval timelines for generic medicines in six countries in the SADC region. For products approved in 2019, it was observed that Namibia had a mean approval time of 890 days, 611 days in Zimbabwe, 589 days in South Africa, 310 days in Mozambique, 218 days in Tanzania and 240 days in Zambia. From this study, it was noted that the mean approval time for Zambia was less as compared with Zimbabwe, South Africa and Mozambique, while Tanzania had the shortest approval timeline. It was noted that the approval for the years 2020 to 2023 increased as compared with the year 2019. A similar study conducted in Brazil looked at the timelines for approving medicines between 2013 and 2016. It was noted that the overall median approval time was 795 days and it was observed that the backlog is a common problem that needs concerted efforts to ensure timely accessibility of medicines to patients [34].

With the onset of COVID-19 in 2019 and 2020, Zambia experienced an increase in the number of vaccines being submitted for approval, demonstrating a need to improve the training of assessors in the evaluation of vaccines and biologicals. To shorten the timelines for these products that are complex in nature there is need to rely on the decision made by the WHO- listed NMRAs. The WHO promotes good reliance practices, which allows an authority to leverage the regulatory work performed by competent regulatory authorities, with the relying authority making an independent final decision [35]. For this to be implemented, there must be trust between the NMRAs and the GBT builds the trust required, strengthened by ensuring that regulatory decisions are based on robust scientific evidence [36]. A study conducted on the use of reliance to review NAS submitted to the South African Health Products Regulatory Authority (SAHPRA) showed that the overall review time was reduced by half as compared to full review [37].

The implementation of GRevP plays a pivotal role in ensuring consistency, predictability, clarity and efficiency in the product review process [38] and the principles of good regulatory practices should be applied in the review of medical products [39]. The quality of the decision made once the product has been reviewed is as important as the review process itself. Despite NMRAs having the same information submitted for registration of products, decisions are not always the same [40]. It is therefore important to improve transparency and accountability in the way decisions are made. This can be improved also through publishing of the assessment and inspection reports.

A well-established quality management system is cardinal in ensuring that the review process is conducted in line with the set standards that will ensure that reviews are carried out in line with GRevPs and the final decision is based on the balance between benefits and harms. The benefit-risk assessment is then communicated to the applicant, patients and the healthcare professionals through public assessment reports [23], although currently the Authority does not publish the summary of evaluation reports. Continuous monitoring of the registration process is critical in ensuring its effectiveness [33]. With the introduction of the electronic integrated management system, the Authority can track applications electronically and reduce the interaction with the applicant.

## Recommendations

The following recommendations were identified from this study. It would be important to:

- Conduct a comparison with similar size stringent regulatory authorities to identify best practices of medicines review including using recognition, reliance and collaboration.
- Publish public assessment reports for all marketing Authorisation applications to enhance transparency.
- Implement a systematic and well-structured quality decision-making practice framework and compare with the regulatory authority of similar size and capacity.
- Establish clear timelines for both regulatory authority reviews and the industry or client time.
- Provide more training to the staff on structured benefit/risk assessment methodologies used in the review process.

## Data Availability

Data is provided within the manuscript.
